# Prediction of radiation pneumonitis with machine learning using 4D-CT based dose-function features

**DOI:** 10.1093/jrr/rrab097

**Published:** 2021-10-27

**Authors:** Yoshiyuki Katsuta, Noriyuki Kadoya, Shina Mouri, Shohei Tanaka, Takayuki Kanai, Kazuya Takeda, Takaya Yamamoto, Kengo Ito, Tomohiro Kajikawa, Yujiro Nakajima, Keiichi Jingu

## Abstract

In this article, we highlight the fundamental importance of the simultaneous use of dose-volume histogram (DVH) and dose-function histogram (DFH) features based on functional images calculated from 4-dimensional computed tomography (4D-CT) and deformable image registration (DIR) in developing a multivariate radiation pneumonitis (RP) prediction model. The patient characteristics, DVH features and DFH features were calculated from functional images by Hounsfield unit (HU) and Jacobian metrics, for an RP grade ≥ 2 multivariate prediction models were computed from 85 non-small cell lung cancer patients. The prediction model is developed using machine learning via a kernel-based support vector machine (SVM) machine. In the patient cohort, 21 of the 85 patients (24.7%) presented with RP grade ≥ 2. The median area under curve (AUC) was 0.58 for the generated 50 prediction models with patient clinical features and DVH features. When HU metric and Jacobian metric DFH features were added, the AUC improved to 0.73 and 0.68, respectively. We conclude that predictive RP models that incorporate DFH features were successfully developed via kernel-based SVM. These results demonstrate that effectiveness of the simultaneous use of DVH features and DFH features calculated from 4D-CT and DIR on functional image-guided radiotherapy.

## INRODUCTION

The treatment of lung cancer via radiotherapy is challenging as numerous nearby organs are at risk, including the trachea, esophagus, heart, brachial plexus and spinal cord, in addition to surrounding healthy lung tissue in both radiosensitive areas and areas critical to patient survival [1]. Irradiated doses are now conformal while maintaining normal tissue tolerance owing to modern advanced radiotherapy technology including intensity-modulated radiotherapy (IMRT) and volumetric-modulated arc therapy (VMAT) [2,3].

In treatment planning stems, the tolerance limit of healthy lung tissues that surround a tumor has been introduced from the hypothesis that lung function is homogeneously localized. Recently, functional images generated by modalities such as hyperpolarized ^3^He-MRI [4], Xenon-CT [5], and nuclear medicine [6] identify lung function profiles as physiological information to be introduced in radiotherapy planning. Studies have demonstrated better correlations between radiation pneumonitis (RP) occurrences and features calculated from functional images [4–6] than those found using conventional dose-volume histogram (DVH) features [7, 8]. Functional image-guided radiotherapy [9, 10], which avoids irradiation to highly functioning lung regions, is gaining attention as a technology that reduces RP through high-spatial resolution physiological information. This radiotherapy, as reported by Yamamoto *et al.* [9, 10], is designed to selectively avoid irradiating highly functional lung regions detected using 4-dimensional computed tomography (4D-CT) and deformable image registration (DIR) while meeting the standard dose limits to critical organs.

Machine learning is one way to cover important topics in radiotherapy, such as predicting toxicities, and several researchers have already reported on this [11–13]. Machine learning considers a large number of variables, so it provides an alternative data-driven approach for predicting common patterns in toxicities resulting from radiotherapy. Our study has demonstrated the effectiveness of the simultaneous use of DVH and dose-function histogram (DFH) features calculated from functional images generated using 4D-CT and DIR by developing a predictive model for RP occurrences.

## METHODS AND MATERIALS

### Patient clinical features and dose-volume histogram features

The committee of the Ethical Review Board (2020-1-1066) of our hospital approved our study. This retrospective study has included 85 patients with non-small cell lung cancer from 2013 to 2019 who received a fractional size of 1.8–3.0 gray (Gy)/fraction (total 60–72 Gy) photon beam radiotherapy and required a minimum of a 6-month follow-up period in our hospital. Treatment was delivered using a 3-dimensional conventional radiotherapy (3D-CRT) technique on 50/85 patients and a VMAT delivery technique on 35/85 patients with or without chemotherapy. For each patient, to consider respiratory motion, the gross internal tumor volume (GITV) was delineated using a set of 4D-CT images comprising 10 respiratory phases sorted via a real-time position management system (Varian Medical Systems, California, USA). The clinical target volume (CTV) was determined by isotropically adding a 5-mm margin to the total volume of each GITV, and the planning target volume (PTV) was determined by expanding the CTV by 5 mm in all directions.

All planned external beam doses were calculated using an analytical anisotropic algorithm or an Acuros XB algorithm equipped with Eclipse TPS (Varian Medical Systems, California, USA). Patient clinical features including age, the Brinkman index, GITV position, and the volume of the GITV were computed to develop a multivariate RP prediction model. According to Vinogradskiy *et al.* [14], GITV positions are expressed as normalized continuous variables from 0 to 1 along upper–lower, anterior–posterior and left–right directions in lung volume.

For a correction of different fractionation schemes, physical dose distributions were first converted into biological equivalent dose in 2 Gy/fraction (EQD_2_) using a linear quadratic model-based calculation with α/β = 3 [15]. DVH features including mean lung dose (MLD), V5Gy (the fractional volume of healthy lung tissues receiving doses exceeding 5 Gy), and V10Gy to V50Gy in 10 Gy increments were tracked from the calculated dose on patient CT images. Patient CT images were acquired using BrightSpeed 16 (GE Healthcare, Milwaukee, Wisconsin, USA) or SOMATOM Definition Flash AS+ (Siemens Healthcare, Erlangen, Germany).

### Dose-function histogram-based features

Dose-function parameters can be evaluated using DFH concepts based on functional lung images that are generated by DIR of the 4D-CT image sets and quantitative image computation. All 4D-CT images applied in this study consisted of 10 respiratory phases. The peak-inhalation image was spatially registered with the peak-exhalation image with nonparametric volume-based DIR using the Elastix open-source toolkit [16]. Parameter sets achieved a spatial accuracy of <1.3 mm [17] for these images. Lung function was quantified with a Hounsfield unit (HU)-based metric [18] and a Jacobian metric [19] per the following equations using registered peak-inhalation and peak-exhalation images. Both metrics are based on the assumption that regional ventilation is proportional to regional volume change, which is supported by the papers that found a reasonable correlation with Xe-CT-measured regional ventilation in sheep [18, 19]. The HU metric is originated from the concept that CT numbers comprise a linear combination of water- and air-like materials (equation 1).

(1) \begin{eqnarray*} && \qquad{V}_{\mathrm{HU}}\left(x,y,z\right)= \\ && \frac{HU_{ex}\left(x,y,z\right)-{HU}_{in}\left\{x+{u}_x\left(x,y,z\right),y+{u}_y\left(x,y,z\right),z+{u}_z\left(x,y,z\right)\ \right\}}{\left[{HU}_{in}\left\{x+{u}_x\left(x,y,z\right),y+{u}_y\left(x,y,z\right),z+{u}_z\left(x,y,z\right)\ \right\}+1000\right]},\nonumber \end{eqnarray*}

where, *HU_ex_*, *u,* and ${HU}_{in}\big\{x+{u}_x\big(x,y,z\big),y+{u}_y\big(x,y,z\big),z+{u}_z\big(x,y,z\big)\ \big\}\ u$ are the peak-exhalation image, displacement vector mapping voxel at the location $\big(x,y,z\big)$ of a peak-inhalation image to the corresponding location of peak-exhalation image, and registered peak-inhalation image, respectively. In principle, by assuming air and tissue densities to be–1000 and 0 HU, respectively, the Jacobian metric is given by (equation 2).

(2) \begin{align*} & {V}_{\mathrm{Jac}}\left(x,y,z\right)= \nonumber \\ &\left\{\left|\begin{array}{ccc}1+\frac{{\partial u}_x\left(x,y,z\right)}{\partial x}& \frac{{\partial u}_x\left(x,y,z\right)}{\partial y}& \frac{{\partial u}_x\left(x,y,z\right)}{\partial z}\\{}\frac{{\partial u}_y\left(x,y,z\right)}{\partial x}& 1+\frac{{\partial u}_y\left(x,y,z\right)}{\partial y}& \frac{{\partial u}_y\left(x,y,z\right)}{\partial z}\\{}\frac{{\partial u}_z\left(x,y,z\right)}{\partial x}& \frac{{\partial u}_z\left(x,y,z\right)}{\partial y}& 1+\frac{{\partial u}_z\left(x,y,z\right)}{\partial z}\end{array}\right|-1\right\} \end{align*}

This metric was calculated by directly tracking the Jacobian of DIR results and is based on the idea that local partial derivatives are related to the volume change of a given voxel. For each patient, the image artifacts on 4D-CT were reviewed, and the manual trimming of central airways and great vessels were performed.

Each ventilation value on the quantified 4D-CT-based lung functional images were converted into a percentile in a manner according to that reported by Vinogradskiy *et al.* [20]. Thereafter, the DFH features including the functional MLD (fMLD; MLD weighed using regional function) and percent lung function receiving 5 and 10–50 at 10 Gy intervals (fV5 and fV10 Gy–fV50 Gy, respectively) were computed using this percentile ventilation values and calculated dose on TPS [21].

### Multivariate analysis with support vector machine

Whole features were converted to Z scores (mean = 0, standard deviation = 1), as was recommended by Kang *et al.* [22]. Converted scores, which are expressed as mean and standard deviation, were compared across each predictor. In this study, a kernel-based support vector machine (SVM) was adopted to the prediction model for RP that was applied previously to radiotherapy [12].

Hyperparameter optimizations and prediction model performance tests were performed through cross-validation [23]. The optimal hyperparameters including C and γ for kernel-based SVM with nonlinear radial basis function (RBF) kernels were determined by Bayesian optimization through 5-fold cross-validation to prevent overfitting in the training partition. In kernel-based SVM, parameter C represents the penalty that regulates the tradeoff between the maximized difference between classes and the minimized misclassification rate in the training set. Parameter γ represents the size of the RBF kernel. Then, in the testing partition generalization performance, that is predictions of a given set of candidate predictors in an external data set, was computed [24].

In this study, 50 prediction models were generated (10 times of cross-validation). Considering the data set size, they were split into two parts (70% for the training partition and 30% for the testing partition) with random sampling in an attempt to balance the class distributions over every cross-validation partitions. Four sets of candidate features were used to determine the capability of DFH features to develop RP prediction models: (i) patient clinical features, (ii) + DVH features, (iii) + DFH features, and (iv) + DVH and DFH features. In this study, MATLAB (MathWorks, Natick, Massachusetts, USA)-based programs were utilized to feature calculation and prediction model development.

## RESULTS

Patient characteristics are summarized in Table 1. Of the 85 patients, 21 (24.7%) determined by a ≥ grade 2 RP by Common Terminology Criteria for Adverse Events (version 4.0) scoring system on the basis of clinical presentation and radiographic findings retrospectively.

**Table 1:** Summary of patient characteristics

Characteristic		*n*	%
Gender	Male	65	76.5
	Female	20	23.5
Delivery technique	3D-CRT	50	58.8
	VMAT	35	41.2
RP classification	≥ Grade 2	21	24.7
	< Grade 2	64	75.3
Smoking history	Yes	70	82.4
	No	15	17.6
COPD	Yes	41	48.2
	No	44	51.8
Fractionation	60Gy/30fr	56	65.9
	66Gy/33fr	19	22.3
	69Gy/23fr	6	7.1
	72Gy/24fr	4	4.7
Chemotherapy	Yes	39	45.9
	No	46	54.1
Regimen of chemotherapy	Cisplatin/vinorelbine	24	61.5
	Carboplatin/paclitaxel or nab-paclitaxel	10	25.6
	Cisplatin/etoposide	2	5.1
	Cisplatin/pemetrexed	1	2.6
	Others	2	5.1

Abbreviations: 3D-CRT = 3-dimensional conventional radiotherapy; VMAT = volumetric-modulated arc therapy; RP = radiation pneumonitis; COPD = chronic obstructive pulmonary disease. Data presented as mean ± standard deviation.

**Fig. 1:**
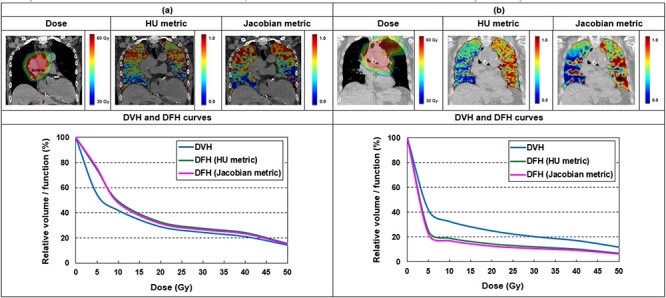
Example for dose distribution, ventilation distribution calculated by HU metric, Jacobian metric and DVH and DFH curves on a representative patient. Abbreviations: HU metric = Hounsfield unit metric*;* DVH *=* dose-volume histogram; DFH = dose-function histogram.

Dose distribution and ventilation distribution computed by the HU metric, Jacobian metric, and DVH and DFH curves for representative patients with ≥grade 2 RP were shown in Fig. 1. As is presented in Fig. 1(a), the patient’s high-function area was located close to the tumor. It was detected by both metrics that the high-function area received treatment delivery. DFH features calculated by the HU metric were 74.5% of fV5 Gy and 32.5% of fV20 Gy, by the Jacobian metric were 75.7% of fV5 Gy and 31.4% of fV20 Gy. A high-function area located far from the tumor, as shown in Fig. 1(b), was also shown by both metrics to have received lower doses (25.2% and 14.5% by the HU metric and 22.4% and 12.6% by the Jacobian metric). Table 2 provides the mean and standard deviation of the patient clinical features, DVH features, and DFH features of the 85 patients calculated, respectively. P-values were computed from Mann–Whitney U test.

**Table 2:** Summary of patient clinical features, DVH and DFH features

Features		Values					*P value*
Age		70.3 ± 10.9					0.32
Brinkman index		667.8 ± 487.7					0.36
GITV location	Upper – lower	0.41 ± 0.10					0.20
	Anterior – posterior	0.56 ± 0.05					0.22
	Left – right	0.49 ± 0.10					0.37
Lungs minus GITV		2906 ± 793 cc					0.33
Features		DVH (%)	*P value*	HU metric (%)	*P value*	Jacobian metric (%)	*P value*
V_5_		41.4 ± 16.20	0.01				
V_10_		29.66 ± 11.16	0.09				
V_20_		20.48 ± 7.94	0.31				
V_30_		14.96 ± 6.99	0.54				
V_40_		10.44 ± 6.10	0.56				
V_50_		6.58 ± 5.05	0.81				
MLD		11.68 ± 3.97	0.22				
fV_5_				41.86 ± 18.23	0.01	41.96 ± 18.49	0.01
fV_10_				29.55 ± 12.46	0.03	29.68 ± 12.80	0.05
fV_20_				20.26 ± 8.79	0.08	20.25 ± 9.02	0.10
fV_30_				14.89 ± 7.68	0.24	14.83 ± 7.84	0.28
fV_40_				10.53 ± 6.71	0.24	10.4 ± 6.85	0.28
fV_50_				6.75 ± 5.55	0.46	6.72 ± 5.89	0.52
fMLD				11.71 ± 4.68	0.05	11.74 ± 5.04	0.05

Abbreviations: GITV = gross internal tumor volume; DVH = dose-volume histogram; DFH = dose-function histogram; HU metric = Hounsfield unit metric. P values were calculated using univariate logistic regression. Data presented as mean ± standard deviation.

The area under curve (AUC) resulting from the different training and testing partitions for 50 developed models is shown in Table 3 for four candidate features, including: (i) patient clinical, (ii) + DVH features, (iii) + DFH features, and (iv) + DVH features and DFH features. The mean difference in AUC for prediction models developed using each feature set between the training partitions and the testing partitions ranged from 0.002 for patient clinical features to 0.049 for + DVH features and + DFH features (as calculated by the Jacobian metric). Developed prediction models achieved performances comparable to results obtained with hyperparameter optimization within the external data set.

**Table 3:** Performance of 50 developed models on training partitions and testing data partitions

	AUC (95% CI)	
Feature sets	Training partitions	Testing partitions
PC	0.55 (0.54-0.57)	0.53 (0.53-0.54)
PC + DVH	0.56 (0.53-0.59)	0.56 (0.54-0.57)
PC + DFH (HU metric)	0.61 (0.6-0.63)	0.60 (0.59-0.61)
PC + DFH (Jacobian metric)	0.63 (0.61-0.65)	0.61 (0.59-0.62)
PC + DVH + DFH (HU metric)	0.74 (0.71-0.76)	0.73 (0.72-0.75)
PC + DVH + DFH (Jacobian metric)	0.74 (0.71-0.77)	0.69 (0.67-0.71)

Abbreviations: AUC = area under curve; PC = patient clinical; DVH *=* dose-volume histogram; DFH = dose-function histogram; HU metric = Hounsfield unit metric; CI = confidence interval.

**Fig. 2:**
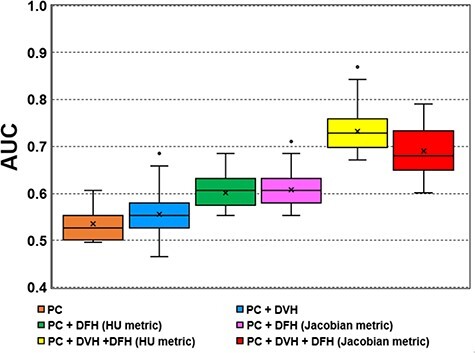
Box plot of the AUC on testing data partitions of 50 developed models. Abbreviations: AUC *=* area under curve; PC = patient clinical; DVH = dose-volume histogram; DFH = dose-function histogram; HU metric = Hounsfield unit metric.

On the testing partition, the median (quartile deviation ranged from 25% to 75%) AUC for the prediction model on + DVH of 0.55 (0.05) was slightly large for the patient clinical prediction model of 0.53 (0.05), as shown in Fig. 2. For the prediction models of + DFH features, on the HU metric and Jacobian metric, the median AUC improved to 0.61 (0.06) and 0.61 (0.05), compared with the + DVH features model, with significant difference (*P* < 1.0^−10^; paired Wilcoxon sing rank test). These results indicate that the DFH features which originated from 4D-CT functional images were deemed effective in developing predictive RP models. Further, + DVH and DFH features models on the HU and Jacobian metrics achieved the median AUC at 0.73 (0.06) and 0.68 (0.08) compared with the + DVH feature model and + DFH features models, with statistically significant difference (*P* < 1.0^−10^), showing that simultaneous use of these features further improved the prediction of RP.

**Fig. 3:**
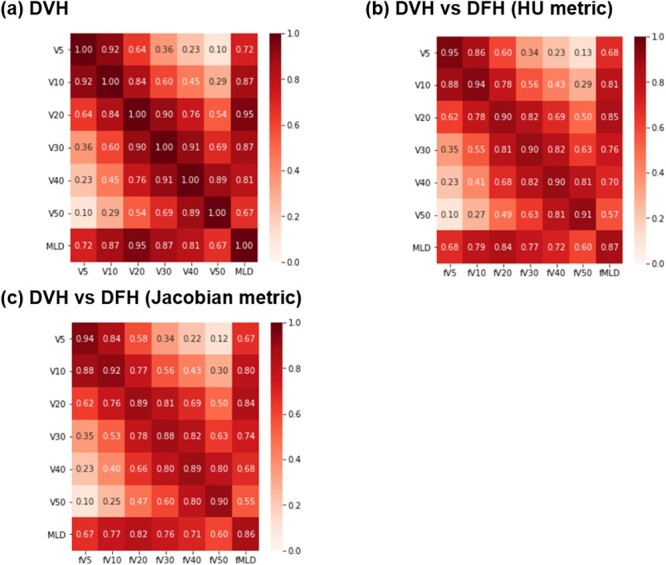
Feature correlation heat map illustrating the correlation between (a) DVH, (b) DVH vs DFH on HU metric, and (c) DVH vs DFH on Jacobian metric. Abbreviations: DVH *=* dose-volume histogram; DFH = dose-function histogram; HU metric = Hounsfield unit metric.

DVH features were strongly positively correlated to each other as is presented in Fig. 3(a). For example, Spearman’s correlations of V20 Gy to V5 Gy and MLD are 0.64 and 0.95, respectively. In contrast, there was a distribution of correlations between DVH features and DFH features. For example, on the HU metric and Jacobian metric, the correlation of V20 Gy decreased to 0.62, 0.62 for fV5 Gy, and 0.85 and 0.84 for fMLD. A comparison between both metrics is shown in Fig. 3(b) and (c). This comparison demonstrates that correlations from DVH features to DFH features on the HU metric were substantially comparable to those on the Jacobian metric.

## DISCUSSION

There has been growing interest in functional image-guided radiotherapy that achieves a reduction in RP by diminishing doses to high-functioning lung areas. Thus, in this study, it was demonstrated that RP prediction evaluated with AUC value in multivariate models with patient clinical and patient clinical + DVH features was improved significantly by adding DFH features computed from 4D-CT and DIR. These results show the fundamental importance of the simultaneous use of both DVH and DFH features in functional image-guided radiotherapy. This simultaneous use of DVH and DFH features supports the involvement of RP prediction in clinical decision-making on treatment planning.

In this study, RP was predicted using machine learning. Machine learning, which offers a data-driven approach to the development of RP prediction models, presents the rules among many features and predicts RP occurrence based on the integration of these features. By adding DFH features calculated using HU and Jacobian metrics, the median AUC for 0.55 on DVH features was improved to 0.61 and 0.61 (P < 1.0−10), respectively. Finally, the patients’ clinical DVH and DFH features reached a mean AUC of 0.73 and 0.68 (P < 1.0−10), respectively. Machine learning demonstrated the potential to predict RP through simultaneous use of multiple features by these successfully developed prediction models.

Machine learning SVM, which is a supervised classification method derived from statistical learning theory, predicts RP by using a hyperplane that maximizes the margin between two classes on multi-dimensional space. Effects on prediction power and robustness of the developed model depends on hyperparameters, including C and γ, to determine hyperplane. Approaches such as a grid-search and random-search, spans huge combinations that require huge calculation costs in the domain in hyperparameter optimization. In general, many modern computations including machine learning are designed with a tradeoff of accuracy for increased performance [25]. The refinement of this tradeoff using the Bayesian optimization as the basis of probabilistic search [26], resulting in RP prediction improvement was given from deciding hyperplane on multi-dimensional spaces consisting of patients’ clinical DVH features and DFH features by searching optimal hyperparameters. Note that building a predictive model, even using Bayesian optimization, is difficult when features with poor predictive power are added to the multidimensional space.

In multivariate analysis, SVM approaches are affected by overfitting that memorizes unavoidable noise on the training partition instead of learning the discipline hidden behind the data. This is one of the major problems in the development of multivariate prediction models. Overfitted models tend to have a decreased generalization performance that conducts predictions on external data sets and have no observations from the training data set [24, 27]. To prevent overfitting and the optimistic estimate of performance, the prediction model in this study was developed from cross-validation [27] while assessing bias risk and clinical usefulness. In the Results section, the prediction model performances for each candidate feature category on the training partition and the testing partition were shown. The mean differences in AUCs for the developed models using each candidate feature set between training partitions and testing partitions as shown in the Results section demonstrate inhibition of overfitting.

Relatively poor discriminative performance of patient characteristics + DVH features (as is shown in Fig. 2) appears to be in conflict with previous reports [28, 29], suggesting the predictive performance of DVH features in RP prediction. When developing the prediction model, it is important to consider the differences in patient characteristics included in the data set. Subsequent effects of dosimetric parameters may be held in the underlying dose distribution which is susceptible to delivery technique differences [30]. Patient cohorts in previous studies [28, 29] mostly received 3D-CRT, in contrast to the cohort in this study (including cases that received VMAT). In fact, AUC value of 0.47 for patient characteristics + DVH features were obtained in a patient cohort including IMRT patients for RP prediction using radiomics features [30]. Therefore, because dosimetric indices or selected model parameters are inconsistent between cohorts, it is not expected that a prediction model applied to a different patient cohort will be able to achieve similar prediction results.

Because our patient cohort did not have enough patients for separate analyses, our prediction models were developed using DFH and DVH features calculated from patients both with and without chemotherapy. The tolerance of OARs to radiotherapy will be lower when chemotherapy is included. The prediction model may perform better when taking the use of chemotherapy into consideration, by separately analyzing patients with and without chemotherapy.

However, machine learning algorithms have shown that this is not always the case. Complex and high-dimensional feature distribution often degrades the performance of learning algorithms because they contain irrelevant and redundant information. Menze *et al.* improved predictive performance while eliminating irrelevant and redundant features in radiotherapy [31]. This study did not eliminate any features because the focus was on the demonstration of RP prediction improvement when DFH features were added to patient clinical and DVH features during prediction model development. The potential for a multivariate model developed with optimal features decided by eliminating irrelevant and redundant features from candidate features, including patient characteristics, DVH features and DFH features, is worth considering.

Results in this study are based on retrospective data collected from a single center. Therefore, further prospective and validation using an external patient cohort is recommended. To date, there are no theories concerning kernel selection for SVM from linear/nonlinear, even though several researchers have already studied this [32]. Therefore, improvements in RP prediction with SVM using RBF kernel in this study can vary. It is thus recommended that kernel selection be completed in a data-dependent way while paying attention to the balance between generalization performance and expression power.

DFH computing has often been performed on advanced non-small cell lung cancer, which had a relatively large, irradiated lung volume and received RP occurrence relatively frequently, compared with early-stage non-small cell lung cancer. In advanced non-small cell lung cancer radiotherapy, a patient whose high-function area is located close to the tumor especially uses DFH features for RP prediction [20]. As a routine use of thoracic radiotherapy, DFH features have the potential to be spread to many clinics. Thoracic radiotherapy routinely uses 4D-CT scans, and thus can be considered free information. In addition, the entire machine learning process in this study, including hyperparameter optimization, can be performed using a general-purpose desktop personal computer. The time to integrate features using machine learning was within 30 min using a desktop computer installed with an Intel core i9-10 920X processor.

The physiological significance of 4D-CT functional image applied DFH computing was researched by Yamamoto *et al.* [6]. The lowest 25th percentile function value on the 4D-CT functional images shows moderate correlations with forced expiratory volume in 1 s (FEV1)/forced vital capacity and FEV1, indicating 4D-CT defect correlated with impaired global lung function. Further, there are moderate dice similarity coefficients between 4D-CT functional images and SPECT defect regions. These correlations may increase by improving 4D-CT artifacts, which is an important source of variations in 4D-CT function imaging. Currently, phase-based sorting applied when generating 4D-CT as a standard technique often resulted in artifacts. Solutions to accelerate the potential for the 4D-CT functional image that correlate to pulmonary function test that anatomic similarity-based sorting to use images with fewer artifacts for analysis [33].

In addressing study limitations, it is important to note that 4D-CT ventilation imaging is undergoing continual improvement, as this is a developing technique [4, 5, 33–35]. The following topics have been studied in the process of generating ventilation imaging: imaging artifact and noise [33], the spatial accuracy of DIR [34], reproductivity [35], the normalizing method, noise reduction filtering and correlations to hyperpolarized ^3^He-MRI [4], Xenon-CT [5] and SPECT [6]. Promising results have been shown for 4D-CT ventilation imaging as a means of imaging regional air volume changes when compared to other ventilation imaging. The predictors derived from the dose and function metrics that were utilized for the RP prediction model assumed that the relationship between the spatial distribution of the dose and high-functioning areas is constant. Previous papers suggest that the reopening of airways (due to tumor shrinkage in response to irradiation) introduces areas of previous ventilation defects that can be reventilated [36]. Notably, 99 mTc-DTPA SPECT ventilation imaging, which is a widely accepted clinical standard method for evaluating regional lung function, has limited image quality due to low resolution and central airway depositions observed frequently in patients with COPD [37]. Therefore, no standard modality provides physiological significance as a ground truth between functional modalities. Continual work to validate functional imaging and measures of lung function is warranted in larger populations.

## CONCLUSION

Irradiation of highly functional lung regions was selectively reduced in functional guided radiotherapy with DFH features while meeting standard dose limits to critical organs quantified by DVH features. This study has demonstrated the effectiveness of the simultaneous use of DVH features and DFH features, calculated from 4D-CT and DIR by developing RP prediction models.
